# New Perspectives in Iron Chelation Therapy for the Treatment of Neurodegenerative Diseases

**DOI:** 10.3390/ph11040109

**Published:** 2018-10-19

**Authors:** Marco T. Nuñez, Pedro Chana-Cuevas

**Affiliations:** 1 Faculty of Sciences, Universidad de Chile, Las Palmeras 3425, Santiago 7800024, Chile; 2 Center for the Treatment of Movement Disorders, Universidad de Santiago de Chile, Belisario Prat 1597, Santiago 83800000, Chile; pedro.chana@usach.cl

**Keywords:** neurodegeneration with brain iron accumulation, iron chelation therapy, multifunctional iron chelators

## Abstract

Iron chelation has been introduced as a new therapeutic concept for the treatment of neurodegenerative diseases with features of iron overload. At difference with iron chelators used in systemic diseases, effective chelators for the treatment of neurodegenerative diseases must cross the blood–brain barrier. Given the promissory but still inconclusive results obtained in clinical trials of iron chelation therapy, it is reasonable to postulate that new compounds with properties that extend beyond chelation should significantly improve these results. Desirable properties of a new generation of chelators include mitochondrial destination, the center of iron-reactive oxygen species interaction, and the ability to quench free radicals produced by the Fenton reaction. In addition, these chelators should have moderate iron binding affinity, sufficient to chelate excessive increments of the labile iron pool, estimated in the micromolar range, but not high enough to disrupt physiological iron homeostasis. Moreover, candidate chelators should have selectivity for the targeted neuronal type, to lessen unwanted secondary effects during long-term treatment. Here, on the basis of a number of clinical trials, we discuss critically the current situation of iron chelation therapy for the treatment of neurodegenerative diseases with an iron accumulation component. The list includes Parkinson’s disease, Friedreich’s ataxia, pantothenate kinase-associated neurodegeneration, Huntington disease and Alzheimer’s disease. We also review the upsurge of new multifunctional iron chelators that in the future may replace the conventional types as therapeutic agents for the treatment of neurodegenerative diseases.

## 1. Introduction

Iron content increases with age in several regions of the brain. Particularly, high levels of non-heme iron are found in the globus pallidus, the red nucleus, substantia nigra, cortex and putamen; in contrast, the iron content of the medulla oblongata does not change with age whereas the iron content of the thalamus decreases from age 30 to 90 [1,2,3]. The causes underlying the increase in brain iron with age remain elusive. It is unclear whether this increase is a reflection of total body iron, since a report shows that non-heme iron in the liver does not change with age [1], although body stores of iron, as determined by circulating ferritin levels, seem to increase with age [4].

Abundant evidence suggests that disturbed iron homeostasis and mitochondrial dysfunction play important roles in the development of an increasing number of neurodegenerative diseases [3,5,6,7,8,9]. The occurrence of high iron content in brain areas susceptible to neurodegeneration, in conjunction with the known ability of iron to generate reactive oxygen species (ROS) and induce the formation of protein aggregates, provides a relevant seed mechanism for downstream events leading to the death of affected neurons. It has been postulated that the high iron–ROS–mitochondrial dysfunction events undergo a positive feedback loop that further fosters oxidative damage, protein aggregation and cell death [3,6,8].

The use of iron chelators for the treatment of systemic diseases such as thalassemia major and hemochromatosis is already a proven therapeutic approach [10]. As a norm, chelator treatment in iron overload patients induces substantial iron excretion and a negative iron balance [11,12,13,14,15]. On the basis of this experience, strategies to stop or slow neurodegenerative process with an iron accumulation component are now being tested in therapeutic trials.

A review on the current evidence of the benefits and drawbacks of iron chelation therapy, and the analysis of new compounds that could be used for the treatment of neurodegenerative diseases, follows.

## 2. Neurodegenerative Diseases with an Iron Accumulation Component

A wide variety of neurological diseases are characterized by the accumulation of iron in different areas of the central nervous system; these diseases include Parkinson’s disease (PD) and other parkinsonisms such as Lewy bodies dementia, progressive supranuclear palsy, corticobasal degeneration [16,17,18,19,20,21], the Westfal variant of Huntington disease [22], Alzheimer’s disease (AD) [23,24,25,26,27], Friedreich’s ataxia [28], pantothenate kinase-associated neurodegeneration [29,30,31] and other neuropathologies associated with brain iron accumulation [32,33,34]. From the pathophysiological standpoint, different mechanisms are observed, so the clinical and therapeutic implications of iron accumulation may be different for each individual disease process [2,35].

There is ample evidence linking iron to the pathology of idiopathic PD. A good review on this subject can be found in the article by Moreau et al. [36]. Iron is particularly abundant in dopaminergic neurons, where it is needed for dopamine synthesis [37] and the production of energy through the electron transport chain [38,39]. In dopaminergic neurons, iron behaves as a double-edged sword since it also participates in the production of the noxious hydroxyl radical during dopamine auto-oxidation. Moreover, the nonenzymatic oxidation of dopamine produces the leukoaminochrome o-semiquinone radical, which reacts with oxygen to generate superoxide anion [40,41,42]. Since under physiological conditions iron reacts with superoxide and hydrogen peroxide [6], it is possible that iron dyshomeostasis plays a fundamental role in mediating oxidative damage in dopaminergic neurons. Indeed, the hydroxyl radical, the most reactive ROS in living matter, is formed by the Fenton reaction (Fe^2+^ + H_2_O_2_ → OH^−^ + HO^•^), a nonenzymatic reaction that obeys mass action law. Hence, there is a direct relationship between the concentration of redox-active iron and the production of hydroxyl radical.

The mechanisms of iron homeostasis that go awry in neurodegeneration form a cutting-edge question in metalloneurobiology [43]. Highly relevant to this point is the mitochondria–iron connection. The iron homeostasis regulator iron regulatory protein 1 (IRP1) is activated in idiopathic PD. Postmortem brain tissue from PD patients displays increased IRP1 activity when compared to tissue from control individuals [44]. Increased IRP1 activity was found also in the ipsilateral ventral mesencephalon of 6-OHDA-treated rats [44]. Studies performed in our laboratory showed that in SH-SY5Y cells, loss of mitochondrial function caused by inhibition of complex I results in decreased Fe–S cluster synthesis and increased IRP1 binding activity, accompanied by increased intracellular iron levels [45]. Further studies revealed that complex I inhibition is associated with increased levels of iron uptake proteins, and decreased levels of the iron efflux transporter ferroportin 1 [46]. Complex I inhibition also results in increased oxidative modifications and increased cysteine oxidation, while IRP1 silencing abolishes the increase in ^55^Fe uptake activity and protects cells from death induced by complex I inhibition [46]. Thus, mitochondrial dysfunction initiates a positive feedback loop that also comprises increasing iron uptake and increased oxidative damage. In this view, iron accumulation, more than a primary cause, seems to be a consequence of other initiation factors.

An attractive hypothesis for the genesis of diseases with a redox-active metal accumulation component is the metal-based neurodegeneration hypothesis [3]. According to this hypothesis, redox-active metal ions like iron and copper generate ROS that cause the peroxidation of membrane phospholipids, which in turn leads to the formation of reactive aldehydes. Reactive aldehydes, together with other ROS, interact with proteins inducing their aggregation, which overwhelms the cellular protein degradation systems, resulting in their accumulation within intracellular inclusion bodies. Accordingly, this hypothesis suggests that protein aggregation occurs downstream of iron or copper dyshomeostasis.

**Figure 1:**
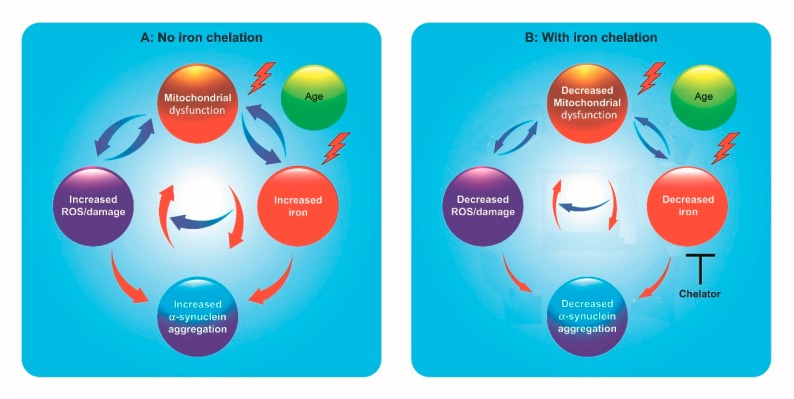
Self-feeding cycles in Parkinson’s neurodegeneration. (**A**) Blue arrows: Mitochondrial dysfunction, caused by internal or external toxins, or derived from genetic factors, results in increased oxidative stress and in decreased synthesis of iron–sulfur clusters, which in turn results in the spurious activation of iron regulatory protein IRP1 and increased iron uptake. Both increased ROS and increased iron produce additional mitochondrial dysfunction through the generation of the hydroxyl radical. Red arrows: Increased ROS and increased iron levels induce α-synuclein aggregation, establishing a positive feedback cycle including mitochondrial dysfunction, further taxing energy production. (**B**) Iron chelation decreases redox-active iron and the production of damaging ROS. The decrease in ROS and redox-active iron results in decreased α-synuclein aggregation. The decrease in ROS and α-synuclein aggregation also results in improved mitochondrial function. Thus, decreasing redox-active iron by chelation slows or stops the process of neuronal death. In this scheme, age is a neurodegeneration factor not influenced by iron chelation.

In the specific case of idiopathic PD, α-synuclein aggregation has been proposed to be a consequence of mitochondrial dysfunction/ROS production [47,48,49] or, inversely, mitochondrial dysfunction has been proposed to be a consequence of α-synuclein aggregation [50,51,52,53]. Thus, it is possible that mitochondrial dysfunction, oxidative stress and α-synuclein aggregation jointly establish a positive feedback cycle that taxes energy production and overloads the protein degradation systems [53,54,55]. This positive feedback concept is further augmented by the observation that iron induces α-synuclein aggregation (see above). Since mitochondrial dysfunction increases iron accumulation, another positive feedback cycle could be formed that includes mitochondrial dysfunction, iron dyshomeostasis and α-synuclein aggregation (Figure 1). It follows that any of the components of these two cycles (mitochondrial dysfunction, oxidative stress, iron dyshomeostasis and α-synuclein aggregation) could initiate these processes. From the therapeutic stand point, multiple-task strategies targeting these events should provide more effective treatment to stop the progression of this disease.

## 3. Clinical Trials Using Iron Chelation

Overwhelming evidence shows that iron accumulation in the brain may contribute to neurodegenerative processes, as shown in recent reviews [7,35,43,56,57]. A tempering view states that cellular iron homeostasis mechanisms are sufficient to limit its toxicity [58]. Nevertheless, excessive iron levels will increase hydroxyl radical generation, for which there are no cellular mechanisms to counteract its noxious effects.

Successful experiences support the use of iron chelation therapy for the treatment of systemic diseases with an iron accumulation component, such as thalassemia major, sickle cell disease and cardiomyopathy associated with hereditary hemochromatosis [59,60,61,62,63,64,65,66,67]. The chelators used in these therapies are deferoxamine, deferasirox, deferiprone and PBT2. Adherence to treatment by patients treated with deferoxamine is low since, as it does not permeate the intestinal barrier, it must be injected. Oral chelators such as deferasirox and deferiprone have better treatment compliance. Deferasirox (Exjade^®^, Novartis Pharma AG, Basel, Switzerland) was the first oral chelator approved for human use in 2005, while Deferiprone (Ferriprox^®^, Apotex Inc., Toronto, ON, Canada) was approved in 2011.

Recently, iron chelation has been introduced as a new therapeutic concept for the treatment of neurodegenerative diseases that have a component of metal ion accumulation [56,68,69,70]. Essentially, the iron chelator should be able to penetrate the cell membranes, as well as the blood–brain barrier, and should have the capacity to extract the chelated iron from the accumulation site and to transfer it to other biological acceptors such as circulating transferrin [68,71]. In addition, small doses of chelators must be used in order to minimize side effects [72,73].

A search in https://clinicaltrials.gov indicated 12 ongoing or finished trials of iron chelation for the treatment of neurodegenerative diseases: four trials for Parkinson’s disease, three for Friedreich’s ataxia, two for amyotrophic lateral sclerosis, one for mild Alzheimer’s disease, one for pantothenate kinase-associated neurodegeneration and one for neurodegeneration with brain iron accumulation. A review on the results of finished trials with published results follows. A number of single-case reports that lack the appropriate controls will not be mentioned here (reviewed by Dusek et al. [70]).

### 3.1. Parkinson’s Disease

A randomized pilot clinical trial tested 40 patients with early-stage PD, tried with deferiprone versus placebo. A dose of 30 mg/kg/day, during a period of six months, produced a decrease in the iron content of the sustantia nigra, evaluated by T3 magnetic resonance [69]. A significant improvement of the motor indicators of the progression of the disease was found. Nevertheless, once the treatment was suspended, iron accumulation reappeared, suggesting a reversal to the pathological state. In a second report of this same study, the usefulness of ceruloplasmin (CP) as a biomarker was emphasized, associating the low activity of this enzyme in Parkinson’s disease with iron overload in the substantia nigra [72]. It was found that after six to 12 months of deferiprone treatment, greater reductions in substantia nigra iron levels and Unified Parkinson’s Disease Rating Scale (UPDRS) motor scores were obtained in patients with higher serum and cerebrospinal fluid levels of CP-ferroxidase activity. A second stage of this project, under the acronym FAIRPARK, intends to enroll 338 participants (https://clinicaltrials.gov/ct2/show/NCT02655315?term=chelation&cond=Parkinson+Disease&draw=2&rank=1).

In another series reported in Clinical Trials by researchers from the Imperial College London, good tolerance to deferiprone was reported in patients with Parkinson’s disease, removing excess iron in dentate and caudate nucleus but with minimal effects on the symptoms of the disease [73].

In summary, the reported results on chelator treatment of Parkinson’s disease discussed above are encouraging in terms of a possible slowdown of the disease progression, granting the development of further long-term trials.

### 3.2. Friedreich’s Ataxia

This genetic disease presents a disorder of iron metabolism associated with chronic inflammation and iron accumulation at the level of the central nervous system, the peripheral system, the myocardium and the endocrine system [74]. It was suggested earlier that a reduction of iron accumulation could be a therapeutic alternative for Friedreich’s ataxia patients [75,76,77]. Initial pilot studies in young patients with no overt cardiomyopathy showed that treatment with 20 to 30 mg/kg/day of deferiprone significantly decreased iron accumulation in the dentate nucleus in the cerebellum, while reducing neuropathy and ataxic gait. Nevertheless, the effects of this chelation therapy on neurological symptoms remained controversial [78]. In a follow-up of this study, a one-year open-label extension, it was determined that deferiprone dosed at 40 mg/kg/day worsened ataxia, as indicated by a four-point mean increase in International Cooperative Ataxia Rating Scale (ICARS) scores in this group (unpublished data, compiled in [79]). As in the first study, results using a dose of 20 mg/kg/day were inconclusive. Nevertheless, a significant reduction in cardiac hypertrophy was an interesting side effect of deferiprone treatment [79].

A study of co-administration of deferiprone and the coenzyme Q10 analog idebenone reported improvement in heart hypertrophy parameters and iron deposits in the dentate nucleus, but no improvements in ICARS [80]. Another study that used deferiprone in association with idebenone also failed to find significant improvement in neurological function. Nevertheless, an improvement in heart hypertrophy was reported as possible [81].

The largest series reported for the treatment of Friedreich’s ataxia enrolled 72 patients, who were treated with varied doses of deferiprone, 20, 40, or 60 mg/kg/day in a six-month Phase-II placebo-controlled trial. The results confirmed the safety of deferiprone at doses lower than 20 mg/kg/day, while the 60 mg/kg/day dose was discontinued due to worsening of ataxia in two patients [82]. Patients receiving 20 mg/kg/day of deferiprone showed a decline in the left ventricular mass index but did not present changes in ICARS scores [82]. The decrease in cardiomyopathy correlated with the decrease of iron in the cardiac muscle.

In a study with 13 Friedreich’s ataxia patients, a triple therapy with deferiprone plus idebenone and riboflavin (both antioxidants) resulted in four patients discontinued due to adverse effects after 15–39 months of therapy. Other parameters, like the annual worsening rate scale, the scale for the assessment and rating of ataxia scores and cardiac function did not present significant changes [83]. The authors concluded that the benefits of this triple therapy are uncertain.

In summary, it has been established that therapeutic doses of deferiprone, 20 mg/kg/day, appear to be safe for long-term use for the treatment of Friedreich’s ataxia. In most trials, this treatment produced some improvement in heart function, but no improvement of the neurological symptoms were apparent. In addition, doses higher than 40 mg/kg/day seemed to worsen the disease. The effects of long-term treatment at low doses on, for example, slowing or stopping disease progression, need to be evaluated.

### 3.3. Neurodegeneration with Brain Iron Accumulation (NBIA) Disorders

Within the spectrum of iron deposition disorders there is a group of genetic diseases that have in common a syndrome of NBIA [32,84,85,86]. These disorders include pantothenate kinase-associated neurodegeneration (PKAN, previously known as Hallervorden-Spatz syndrome) [87,88,89,90], PLA2G6 calcium-independent phospholipase A2 (PLAN) [91,92], infantile neuroaxonal dystrophy (INAD) [93,94], mitochondrial membrane protein-associated neurodegeneration (MPAN) [95,96,97,98], beta-propeller protein-associated neurodegeneration (BPAN) [99,100], CoA synthase protein-associated neurodegeneration (CoPAN) [101,102,103], fatty acid-2 hydroxylase-associated neurodegeneration (FAHN) [104,105], Kufor–Rakeb disease [106,107,108], aceruloplasminemia [109,110] and neuroferritinopathy [111,112]. Of these diseases, iron chelation therapy has been tried in PKAN patients.

The PKAN neurodegenerative condition is characterized by the presence of iron deposits at the level of the basal ganglia, currently detected by MRI [86,113,114]. There have been several trials oriented to the use of deferiprone for the treatment of PKAN patients [115,116,117,118]. In all trials, deferiprone treatment decreased iron accumulation in the ganglia, in addition to an improvement in the Unified Parkinson’s Disease Rating Scale [115,116,117,118]. Although the series is still too small to establish definitive conclusions, iron chelation may be a therapeutic option for the treatment of PKAN. Additionally, in a downside, treatment of a single BPAN patient with deferiprone had to be interrupted because of worsening of the parkinsonian symptoms [116].

### 3.4. Huntington Disease

In a study with early/mid-stage Huntington disease patients, patients were subjected to daily doses (250 mg or 100 mg) of 5,7-dichloro-2-[(dimethylamino)methyl]quinolin-8-ol (PBT2) or placebo [119]. After treatment for 26 weeks with this iron chelator, none or marginal improvements were found in cognitive tests. The authors concluded that PBT2 was generally safe and well tolerated, but the evaluation of potential benefits remains uncertain and in need of further studies.

### 3.5. Alzheimer’s Disease

In an initial Phase-II trial with Alzheimer’s disease patients, treatment with the iron–copper chelator clioquinol resulted in stabilization of Alzheimer’s Disease Assessment Scale scores, compared to placebo-treated controls. In addition, plasma Aβ1–42 levels declined in the clioquinol-treated group [120]. In a subsequent Phase-II double-blind trial, it was found that patients treated with doses of 250 mg PBT2 exhibited a significant reduction in cerebrospinal fluid Aβ1–42 concentration compared with those treated with placebo [121]. In addition, PBT2-treated patients showed significant improvement in executive function and cognition tests. No serious secondary effects were reported by patients receiving PBT2. The authors concluded that the findings of this study support larger-scale testing of PBT2 in Alzheimer’s disease patients.

## 4. Potential Risks of Iron Chelation Therapy

Iron is vital to life, participating as an essential cofactor in essential cellular processes that include oxygen transport, energy production, DNA synthesis, and a myriad of hydrolysis and redox reactions [122]. Thus, therapeutic iron chelation may affect not only the intended target but also processes that are essential for cell function, generating cases in which “the cure is worse than the disease”. Indeed, excessive iron depletion could result in cognitive decline, by decreasing the activity of enzymes and iron-containing complexes [122,123] and the synthesis of neurotransmitters, such as dopamine, norepinephrine and serotonin [37,124,125,126]. Fortunately, cognitive decline has not been reported in clinical chelation trials for neurodegenerative diseases using therapeutic concentrations of deferasirox, deferiprone, PBT1 or PBT2 [115,116,117,118,119,121,127,128].

In thalassemia patients treated with high concentrations of deferiprone (75–99 mg/kg/day [129]), the most frequent adverse effects were arthritis, nausea and, more critically, agranulocytosis and neutropenia [130,131,132,133], whereas deferasirox was generally well tolerated, with mild gastrointestinal adverse effects [12,134]. A recent meta-analysis, which analyzed results from 34 studies with a total of 2040 young patients with hemoglobinopathies, found increased transaminase adverse effects (between 3.9% and 31.3% in the different studies) and gastrointestinal complaints for both deferiprone (3.7–18.4%) and deferasirox (5.8–18.8%) [135]. Other effects included arthritis, nausea and, most seriously, agranulocytosis in 0.6% to 4% of patients. This work concluded that there may be few but serious adverse reactions in performing iron chelation therapy with these chelators.

Another potential problem with iron chelation therapy is the depletion of other essential metal ions, in particular Zn and Cu [131,136,137]. Accordingly, in a clinical trial of thalassemia with deferiprone, four of eight patients who received treatment for one year showed increased excretion of Zn in the urine and subnormal values of zinc in the serum, associated with areas of dry skin and itching. These symptoms were resolved with zinc supplementation [136].

There are no reports in the literature indicating that iron chelation therapy causes copper depletion. This is possibly because intracellular copper is not free but it is strongly bound to chaperones [138,139].

From the previous analysis, it is clear that the concentration of the iron chelator should be finely tuned to achieve maximal effectiveness in removing excess redox-active iron, and at the same time avoiding toxicity and other side effects.

## 5. New Multifunctional Iron/Copper Chelators with Therapeutic Capacity

The limited success of metal chelation therapy trials using deferiprone raises the need for novel multifunctional agents, which in addition to decreasing iron accumulation, will have the capacity to interfere with two or more symptoms of a given disease, thus improving the possibilities of stopping, and eventually reversing, the neurodegenerative process.

The question arises on what are the characteristics of an ideal compound for the therapeutic treatment of the vicious cycle of mitochondrial dysfunction–iron accumulation–oxidative damage–protein aggregation in neurodegenerative diseases (Figure 1). These basal properties include: (1) effectiveness via oral administration, which implies crossing the intestinal and blood–brain barrier without modification of its therapeutic properties. Compliance of treatment is better with oral drugs than those injected on a daily basis during long-term treatments [140]; (2) low molecular weight and high membrane permeability. The compound must fulfill the rule of 5 of Lipinski, an empirical model that allows one to evaluate qualitatively how a chemical compound could be absorbed once it is orally ingested as a medicine [141]. Desirable additional properties include (3) free radical quenching capacity independent of its iron chelation capacity. Free radical quenching should decrease lipid peroxidation, thus decreasing ferroptosis events [142]; (4) to undergo mitochondrial accumulation. The reason for this last property is that both high concentrations of ROS and high concentrations of iron coexist in the secluded space of the mitochondrion, which makes this organelle particularly prone to oxidative damage [57,143,144]; (5) to have intermediate (µM) affinity and high specificity for iron, consequent with chelation of the redox-active (labile) iron pool, estimated to be in the micromolar range [134,135]; and (6) in addition, the chelator must have “shuttle” capacity, that is, the capacity to deliver excessive intracellular iron to innocuous acceptors such as transferrin or ferritin [145].

During the last two decades, several multifunctional agents (MFAs) have been reported to be effective in experimental models of Alzheimer’s or Parkinson’s disease. These agents are in an experimental phase, with no clinical trials associated with them. Table 1 shows the basic characteristics of these agents, including their metal chelation characteristics, their capacity to act as antioxidants/free-radical scavengers, their route of administration and blood-brain barrier permeability. We will review next the characteristics of MFAs that could become effective therapeutic agents.

### 5.1. Epigallocatechin-3-Gallate (EGCG)

The pioneering work of Youdim’s group at Technion resulted in two of the best-characterized MFAs: EGCG, a component of green tea, and M30, an *N*-propargyl-8-hydroxyquinoline hybrid.

Epidemiological reports associate tea consumption with positive effects in the central nervous system function, such as reduced dementia incidence, delayed PD onset and diminished cognitive impairment in the elderly (reviewed in [171]). Additional studies have shown the neuroprotective effect of EGCG in the MPTP model of PD [146,149,150,172,173]. However, this neuroprotective effect may be secondary to the inhibition of the dopamine transporter (DAT) by EGCG [148,174]. Indeed, MPTP is metabolized to 1-methyl-4-phenylpyridinium (MPP+) by MAO-B in astrocytes; after release from astrocytes, MPP+ is transported into dopaminergic neurons via DAT [175,176]. Thus, ablation, or inhibition, of DAT results in neuroprotection against MPTP/MPP+ [177,178].

The protection exerted by EGCG probably involves direct scavenging of ROS such as superoxide, hydrogen peroxide and nitric oxide [179,180,181]. Nevertheless, the EGCG antioxidant effect is observed only at low concentrations. In-vitro studies show that high concentrations (10–100 µM) of EGCG actually prompt pro-oxidant effects [182,183,184,185,186]. In rat hippocampal neurons, EGCG causes elevation of intracellular calcium and ROS in a dose-dependent way [185,187]. Downstream, high calcium/ROS levels were associated with reduction in the Bcl-2/Bax expression ratio, reduction of the mitochondrial membrane potential and apoptotic cell death [185,188].

In addition, EGCG has anti-inflammatory properties that may result in neuroprotection [189,190,191]. The administration of EGCG to rats subjected to restraint-induced stress improved open-field and step-through behavioral tests, through a mechanism that involves the restoration of PKCα and ERK1/2 expression, which were diminished by stress [192]. Furthermore, EGCG supplementation restores the production of ATP and the expression of the peroxisome proliferators-activated receptor-γ coactivator-1α (PGC-1α), a key regulator of energy production. The authors concluded that EGCG-mediated protection against stress-induced neural injuries is mediated by a PKCα and ERK1/2 signaling pathway linked to PGC-1α-mediated ATP production.

As shown in Table 1, EGCG is a rather promiscuous metal ion chelator, with affinity towards Cu, Fe, Al and Mn. Since Fe, Cu and Mn are redox-active, EGCG should decrease their redox cycling, thus decreasing the production of ROS.

In summary, EGCG has demonstrated antioxidant, anti-inflammatory and metal-binding properties that may be useful in the prevention of neuronal death. However, EGCG metal ion selectivity is poor, and high concentrations of EGCG promote oxidative damage and induce calcium upsurges that may end up in apoptotic cell death. The sum of these characteristics advises against the use of EGCG as an MFA in disease treatment.

### 5.2. MAO-B Inhibitor Hybrids

M30 was one of the first chelators designed with the purpose of multifunctionality, by linking the Fe^3+^ chelator 8-hydroxyquinoline to a propargyl group with MAO-B inhibition activity through a methyl–amino–methyl bridge [151]. M30 has been used successfully as a neuroprotective agent in models of PD, AD and ALS. In vivo, it is effective when given orally so most probably it crosses the intestinal and blood–brain barriers without loss of activity.

Multiple reports have shown the neuroprotective capacity of M30 in the MPTP model of PD. Since MPTP is metabolized to its active form MPP+ by MAO-B, and M30 inhibits the activity of MAO-B, an important caveat for the neuroprotection effect of M30 in the MPTP model is that it could be a reflection of deficient MPP+ formation.

Numerous effects, from antiapoptotic to neuritogenic, can be ascribed to the iron chelation capacity of M30 [193,194]. A major breakthrough was the finding that M30 activates the transcription factor HIF-1α signaling pathway [195]. In fact, M30 stabilizes HIF-1α, by inactivating the prolyl hydroxylase that initiates its degradation, the activity of which depends on oxygen and Fe [196]. The stabilization of HIF1α results in the increased transcription of HIF-1α-dependent genes, including vascular endothelial growth factor, erythropoietin, enolase-1, p21 and tyrosine hydroxylase. In addition, M30 also increases the expression levels of the transcripts of BDNF, GDNF and GAP-43 [195], and induces the expression of the antioxidant enzymes catalase, superoxide dismutase 1 (SOD1) and gGPx [152]. Immunoblot studies showed that M30 also enhances the phosphorylation of PKC, MAPK/ERK kinase, PKB/Akt and GSK-3β [152]. VAR10303, a derivative of M30, demonstrated properties similar to M30 [154], and was successfully tested in the SOD1(G93A) mice model of amyotrophic lateral sclerosis [197,198].

Discarding an effect of the propargyl moiety of M30 on gene expression, it is possible that the remarkable properties of M30 should be common to many iron chelators with the capacity to reach the brain at therapeutic doses. Importantly, the activation of HIF1α by iron chelators opens a wide field of expectations for iron chelation therapy, evolving from merely inhibition of ROS production, which still holds, to the putative activation of neuroregenerative pathways.

### 5.3. Glucose Hybrids

A novel approach in the design of MFAs was the association of metal chelator moieties to glucose, under the consideration that these agents would be preferentially incorporated into the brain given the high density of hexose transporters (GLUTs) at the blood–brain barrier [199,200]. Once in the brain, these hybrids should be taken up by neurons and astrocytes, considered the cells of major energy consumption. Since astrocytes display preferential transport and metabolism of glucose compared to neurons [201], it would be expected that metal-hexose MFAs should preferentially accumulate in astrocytes. The hydroxypyridinone glycoconjugates H2GL^1^ and H2GL^2^ demonstrated substantial affinity for Cu^2+^ and Zn^2+^, and both agents decreased Aβ1–40 aggregation induced by Cu and Zn. They also demonstrated antioxidant activity, probably through their phenolic moieties, capable of quenching free radicals by a hydroquinone/quinone conversion [155]. No in-vivo testing of these agents was reported, so their putative Fe^3+^-chelating and blood–brain barrier permeability properties remain undetermined.

### 5.4. Acetyl Cholinesterase Inhibitor Hybrids

Under the concept that inhibition of acetylcholinesterase (AChE) increases acetylcholine at cholinergic synapses, thus reducing the cognitive deficit [202,203], an approach for the putative treatment of AD is the design and synthesis of MFAs with AChE inhibition activity. This particular strategy is common for MFAs with a tacrine (a cholinesterase inhibitor) component [156,157,158,159] (Table 1). None of these compounds have been tested for brain permeability, although tacrine derivatives, without an iron chelator component, are brain-effective under intravenous and intranasal administrations [204]. At this stage, tests of effectiveness in animal models of AD are needed for further evaluation of their putative usefulness.

### 5.5. Dopamine Receptor Agonist Hybrids

19a, 19b and D-607 are dopamine D2/D3 receptor agonists and metal chelator hybrids, designed under the notion that D2/D3 receptor agonists have been used for the treatment of both motor and psychiatric syndromes in PD [179]. The three compounds demonstrated iron chelation and antioxidant capacity in vitro [180,181]. In addition, 19b partly reversed hypolocomotion in reserpinized rats, and reduced the rotational activity in a 6-OHDA/apomorphine model, thus demonstrating in-vivo neuroprotective activity [181]. D-607 was the product of a further D2/D3 agonist/chelator development by the same research group. The chelator moiety of D-607 was changed from 8-OH quinolone, a Fe^3+^ chelator, to bipyridyl, a Fe^2+^ chelator. D-607 was shown to suppress retinal degeneration in a Drosophila melanogaster PD model that expresses α-synuclein A30P, a PD-causing variant of the protein [180]. In addition, D-607 was shown to confer significant neuroprotection in the mouse MPTP PD model under chronic MPTP administration [180]. Overall, the published evidence points to D-607 as a putative candidate for the pharmacological treatment of PD. A drawback is that D-607 is administrated intraperitoneally, and not orally, which may decrease hypothetical patient compliance.

### 5.6. Curcumin Hybrids

Curcumin analogs are proposed as potential anti-AD drugs, based on the radical scavenger [182,183] and metal chelator [184,185] properties of curcumin. Curcumin analogs A1–A10 were tested for their radical quenching activity and their ability to reduce metal-induced amyloid-beta aggregation [186]. A1, A2, A3 and A4 presented good radical quenching capacity in SH-SY5Y cells while the capacity of A5, A6, A7, A8, A9 and A10 was weak. All analogs, with the exception of A7, A8 and A10, presented the capacity to diminish amyloid beta self-aggregation at IC50s similar to curcumin. The authors reported Fe and Cu chelation capacity by A4 based on the red shift of absorbance peaks at 267 nm and 427 nm. Nevertheless, A4 lacks the two adjacent ketone groups responsible for metal binding by coumarin [184] and it does not have putative metal binding groups in its structure, so the mechanism of iron binding is not apparent. No in-vivo studies were reported for these curcumin analogs.

### 5.7. Benzothiazole–3-Hydroxy-4-Pyridine Hybrids

Another approach for MFAs is the design of constructs of benzothiazole and 3-hydroxy-4-pyridine connected by a variable linker [83]. Given its hydrophobicity, benzothiazole has strong affinity for amyloid plaques [84,85] while the 3-hydroxy-4-pyridine moiety (deferiprone) has strong Fe^3+^ chelation capacity. The linker between these two moieties was modelled in order to obtain AChE inhibitory capacity [83]. Of the tested compounds, 2a and 2d formed Fe^3+^ chelates with affinities similar to deferiprone. The chelators displayed significant antioxidant properties, with compounds 1a, 1b, 1c and 2d having significant AChE inhibitory activity. Accordingly, these same hybrids presented good inhibitory capacity towards Aβ42 self-aggregation, mostly above 40%. In addition, hybrid 2d inhibited zinc-induced Aβ1–42 aggregation [83]. Overall, the hydroxypyridinone–spacer–benzothiazole hybrids appear as good candidate drugs for the treatment of AD, but in-vivo testing is needed before further development.

### 5.8. MAO-B Inhibitors

On the basis of its moderate success in AD treatment [187], the MAO-B inhibitor selegiline has been used for the design of MFAs directed to the treatment of AD [188,189]. The most promising compounds are selegiline–clioquinol hybrids, which combine MAO-B inhibition with metal chelation capacity. Selegiline–clioquinol hybrids tested in vitro demonstrated inhibition of Cu-induced Aβ1–42 aggregation, antioxidant activity and Cu^2+^, Fe^2+^ and Zn^2+^ chelation capacity [189]. Like other MFAs in a proof-of-concept step, demonstration of in-vivo effectiveness is needed.

### 5.9. Histamine H3 Receptor Antagonists

A different approach in the design of MFAs was the design of a histamine H3 receptor antagonist, 1-phenyl-3-hydroxy-4-pyridinone, and the 3-hydroxy-4-pyridinone iron chelator moiety of deferiprone [190], under the rationale that blocking the action of presynaptic H3 receptors produces increased secretion of histamine and other excitatory neurotransmitters [191]. H3 antagonist treatment results in modest effects on cognitive function [192]. The most promising compound, 5c, displayed H3 receptor antagonistic activity, free radical scavenging capacity, copper and iron chelation, and inhibition of self- and Cu^2+^-induced Aβ1−42 aggregation [190]. After intraperitoneal administration to Sprague Dawley rats, compound 5c demonstrated good blood–brain barrier penetration. In conclusion, the histamine H3 receptor antagonist–iron chelator hybrid 5c is brain-permeant and possesses four functions applicable for the treatment of AD, which makes it a good therapeutic candidate.

### 5.10. Coumarin Hybrids

Two coumarin derivatives have been proposed as candidate drugs for the treatment of neurodegenerative conditions with an iron accumulation component [193,194], based on the known qualities of hydroxycoumarins as free radical quenchers [195] and metal chelator [196,197] agents. DHC12 is a 7,8-dihydroxycoumarin with an amino substituent group at position four of the coumarin ring [193]. The molecule is small and quite simple; nevertheless, it has interesting neuroprotective features. DHC12 exhibited metal binding capacity for Fe^2+^ and Cu^2+^. DHC12 distributed to mitochondria, where it chelated the mitochondrial and cytoplasmic labile iron pool. In a cell model of PD, DHC12 protected cells from plasma membrane and mitochondrial oxidative damage. Oral administration of DHC12 protected sustantia nigra neurons in the MPTP model of PD. On the whole, DHC12 emerges as a good candidate for further development as a PD treatment drug.

CT51 is a hybrid of 7-hydroxycoumarin linked through an acetomethyl group to tris(hydroxymethyl)aminomethane (tris). The hydroxyl group in the coumarin ring quenches free radicals [195] while the three hydroxyl residues of tris provide metal binding capacity [198]. In vitro, CT51 exhibited selective Fe^2+^ and Fe^3+^ binding with no apparent interaction with Cu^2+^, Zn^2+^ and other divalent cations. It also demonstrated free radical quenching capacity superior to Trolox. Interestingly, cyclic voltammetry analysis revealed irreversible binding of Fe^3+^ to CT51, an important finding since stopping Fe^2+^/Fe^3+^ cycling in cells should prevent hydroxyl radical production fostered by oxygen and intracellular reductants [8]. In SH-SY5Y cells, CT51 distributed to both mitochondria and cytoplasm bound iron reversibly and protected against rotenone-induced oxidative damage, while in primary hippocampal neurons, CT51 largely prevented the increase in intracellular calcium levels produced by an agonist of redox-sensitive RyR channels [194]. These capacities so-far demonstrated make CT51 a good therapeutic candidate for the treatment of PD, although in-vivo efficacy needs to be demonstrated.

## 6. Conclusions

The aging of the world population introduces an ever-increasing burden of neurodegenerative diseases on public health systems worldwide. Among these diseases, Parkinson’s, Alzheimer’s and other diseases with an iron accumulation component are at the top of the list. Based on initial trials using the iron chelators deferiprone and PBT2, the metalloneurobiology community has reached the conclusion that therapies targeted to decrease the iron content in specific areas of the brain is a viable course of action to slow or stop the progress of these diseases.

Given the multifactoriality of the neurodegenerative process, the use of multifunctional iron chelators is a promising developmental avenue. As discussed in the text, additional properties of future iron chelator drugs should comprise high selectivity for iron, free radical quenching capacity, mitochondrial distribution and the capacity to block protein aggregation. Several of the compounds now in experimental stages have one or more of these additional characteristics. Let us hope that further research will provide treatments that are both effective and affordable for public health systems.

## Figures and Tables

**Table 1:** Iron chelators with multifunctional characteristics.

Compound	Properties/Characteristics	Metal Specificity	In-Vivo Testing	Route of Administration	Brain Permeability	Disease Model	References
EGCG	Metal chelation; antioxidant; neuroprotective; activation of cell survival genes.	Cu^2+^; Fe^3+^; Al^3+^; Mn^2+^	Yes	Intraperitoneal; Oral	Yes	PD, AD	[146,147,148,149,150]
Hydroxyquinoline‒propargyl hybrids M30, VAR10303	Metal chelation; MAO-B inhibition; antiapoptotic; activation of cell survival genes; neuroprotective; neuritogenic.	Fe^3+^ > Cu^2+^ > Zn^2+^	Yes	Oral	Yes	PD, AD, amyotrophic lateral sclerosis	[151,152,153,154]
Hydroxypyridinone glycoconjugates H_2_GL^1^, H_2_GL^2^	Metal chelation; reduction of amyloid-beta aggregation	Cu^2+^ > Zn^2+^	No	Not tested	Not tested; probably yes	AD	[155]
Bis-tacrine hybrids	Metal chelation; AChE inhibition; reduction of amyloid-beta aggregation	Cu^2+^	No	Not tested	Not tested	AD	[156]
8-OH-Quinoline‒tacrine hybrids	Metal chelation; AChE inhibition	Cu^2+^	No	Not tested	Probably yes	AD	[157]
Benzylamine‒tacrine hybrids	Metal chelation; AChE inhibition; inhibition of amyloid-beta aggregation; moderate antioxidant activity	Cu^2+^; Fe^2+^; Zn^2+^	No	Not tested	Not tested	AD	[158]
Phenyl–benzimidazole‒tacrine hybrid	AChE inhibition; metal chelation; inhibition of Cu-induced amyloid-beta aggregation; free radical scavenger	Cu^2+^; other metals not tested	No	Not tested	Not tested	AD	[159]
Coumarin‒tacrine hybrid	Metal chelation; AChE inhibition; inhibition of amyloid-beta aggregation; free radical scavenger	Cu^2+^; other metals not tested	No	Not tested	Not tested	AD	[160]
Piperazine–8-OH-quinolone hybrids	Metal chelation; dopamine D2/D3 receptor agonists; hydroxyl radical scavenger	Fe^2+^; Fe^3+^	Yes	Subcutaneous	Yes	PD	[161]
Dipyridyl‒D2R/D3R agonist hybrids	Metal chelation; dopamine D2/D3 receptor agonist; antioxidant; neuroprotective	Fe^2+^ >>> Fe^3+^	Yes	Intraperitoneal	Yes	PD	[162,163]
Curcumin hybrids	Metal chelation; antioxidant activity; reduction of amyloid-beta aggregation	Cu^2+^; Fe^2+^	No	Not tested	Not tested	AD	[164]
Benzyl–indanone hybrid compound 41	Metal chelation; antioxidant activity; AChE inhibition; inhibition of amyloid-beta aggregation	Cu^2+^	No	Not tested	Not tested	AD	[165]
Benzothiazole–linker–pyridinone hybrids	Metal chelation; antioxidant activity; AChE inhibition; inhibition of amyloid-beta aggregation	Fe^3+^	No	Not tested	Probably yes	AD	[166]
Clioquinol‒selegiline hybrids	MAO-B inhibition; metal chelation; antioxidant activity	Cu^2+^; Fe^2+^; Zn^2+^	No	Not tested	Probably yes	PD	[167]
Deferiprone‒H3 receptor antagonist hybrid C5	H3R inhibition; metal chelation; antioxidant activity; reduction of amyloid-beta aggregation	Cu^2+^∼ Fe^2+^ >>> Zn^2+^	Yes	Intraperitoneal	Yes	AD	[168]
7,8-Dihydroxycoumarin derivative DHC12	Metal chelation; MAO-B inhibition; mitochondriotropic; free radical scavenger; neuroprotective	Cu^2+^∼ Fe^2+^ > Zn^2+^ > Fe^3+^	Yes	Oral	Yes	PD	[169]
Coumarin–tris hybrid CT51	Metal chelation; impedes Fe^2+^/Fe^3+^cycling; antioxidant; mitochondriotropic; calcium upsurge blocker	Fe^2+^ > Fe^3+^	No	Not tested	Not tested	PD	[170]
